# Paradoxical worsening of seizure activity with pregabalin in an adult with isodicentric 15 (IDIC-15) syndrome involving duplications of the GABRB3, GABRA5 and GABRG3 genes

**DOI:** 10.1186/1471-2377-13-43

**Published:** 2013-05-10

**Authors:** Alessandro Di Rocco, Andrea Loggini, Maja Di Rocco, Pietro Di Rocco, Roger P Rossi, Giorgio Gimelli, Carl Bazil

**Affiliations:** 1 New York University School of Medicine, New York, NY, USA; 2 University of Genoa School of Medicine, Genoa, Italy; 3 Giannina Gaslini Institute, Genoa, Italy; 4 Formerly Giannina Gaslini Institute, Genoa, Italy; 5 Robert Wood Johnson School of Medicine and Johnson Rehabilitation Institute, Edison, NJ, USA; 6 Columbia University College of Physicians and Surgeons, New York, NY, USA; 7 Department of Neurology, NYU Langone Medical Center, New York University School of Medicine, 145 E 32nd Street, New York, NY 10016, USA

**Keywords:** IDIC-15, GABA receptors, Pregabalin, Seizures, Lacosamide

## Abstract

**Background:**

Isodicentric 15 syndrome (IDIC-15) is due to partial duplications of chromosome 15 that may includes the q11–13 region that includes genes encoding the α5 (GABRA5) and β3 - γ3 (GABRB3) receptor subunits. The disease causes intellectual and physical developmental delay, seizures, intellectual disability and behavioral disorders that may be related to abnormal GABA receptor function and morphology. Seizures are often severe and may be refractory to treatment. There are however no specific guidelines for the treatment of the seizures and it is unknown whether drugs that affect the GABAergic system have a different effect in IDIC-15 seizures.

**Case presentation:**

We report the case of an adult individual with IDIC-15 whose complex-partial seizures worsened dramatically after the introduction of pregabalin, with increased seizure frequency, frequent generalization, and appearance of new seizure pattern. Her cognitive function and verbal skills also worsened during treatment with pregabalin. Her seizures and cognitive skills quickly improved after pregabalin was discontinued and treatment with lacosamide started.

**Discussion:**

As her genetic testing confirmed that her region of duplication included GABA receptor encoding genes, it is plausible that the worsening of seizures were due to induction of an abnormal GABAergic response to pregabalin.

**Conclusion:**

As her genetic testing confirmed that her region of duplication included GABA receptor encoding genes, it is plausible that the worsening of seizures were due to induction of an abnormal GABAergic response to pregabalin.This case may help define proper therapeutic strategies for the treatment of IDIC-15 associated seizures.

## Background

Different rearrangements may occur in the chromosome region 15q11–13, which is quite unstable because of repeated DNA elements. The rearrangements include deletions associated either with Angelman syndrome or with Prader-Willi syndrome, translocations, inversions and supernumerary marker chromosomes formed by the inverted duplication of proximal chromosome 15. Interstitial duplications, triplications and balanced reciprocal translocations are quite rare while the inversion duplication 15 or idic15 is the most common of the extra structurally abnormal chromosomes. The duplication extension may be variable and may contain or not the Prader-Willi/ Angelmann syndrome critical region q11–13.

Isodicentric 15 syndrome (IDIC-15) is caused by partial duplications of chromosome 15, usually including the q11–13 region that is rearranged as a supernumerary pseudodicentric chromosome or more rarely as an interstitial duplication [1,2]. The typical phenotype includes physical and intellectual developmental delay with intellectual disability, seizures, behavioral abnormalities often of the autistic spectrum, and minor facial dysmorphism [3,4]. The significant variability of the severity of the syndrome can be attributed to the length of the chromosomal duplication, gene dosing effect and epigenetic mechanisms [2,5,6]. There are very few long-term observations that reach into mature age and it is unknown whether, age-related neurological problems manifest in any specific manner in people with IDIC-15, as it occurs in other chromosomal diseases [3].

Seizures are very common in children and adults with IDIC-15. A wide spectrum of seizure disorders have been reported, including absence seizures, myoclonic epilepsy, complex partial seizures, generalized tonic-clonic seizures, and Lennox-Gastaut like-syndrome. Not uncommonly, affected individuals have complex epileptic patterns that are refractory to treatment [7-10]. Although the mechanism has not been firmly identified, seizures may be due to abnormal γ-aminobutyric acid (GABA)-A receptor configuration, as the affected region on chromosome 15q includes genes encoding the α5 (GABRA5) and β3 - γ3 (GABRB3) receptor subunits [11-13]. There are no established guidelines for the treatment of epilepsy in IDIC-15, nor is it known whether the GABA receptor abnormalities affect the response to anti-epileptic drugs, or if the seizure disorder evolves with age.

We report severe paradoxical worsening of epilepsy in an adult woman with IDIC-15 with complex partial seizures and secondary generalization who had been treated with Pregabalin and a dramatic improvement after the drug was discontinued.

## Case presentation

**Figure 1:**
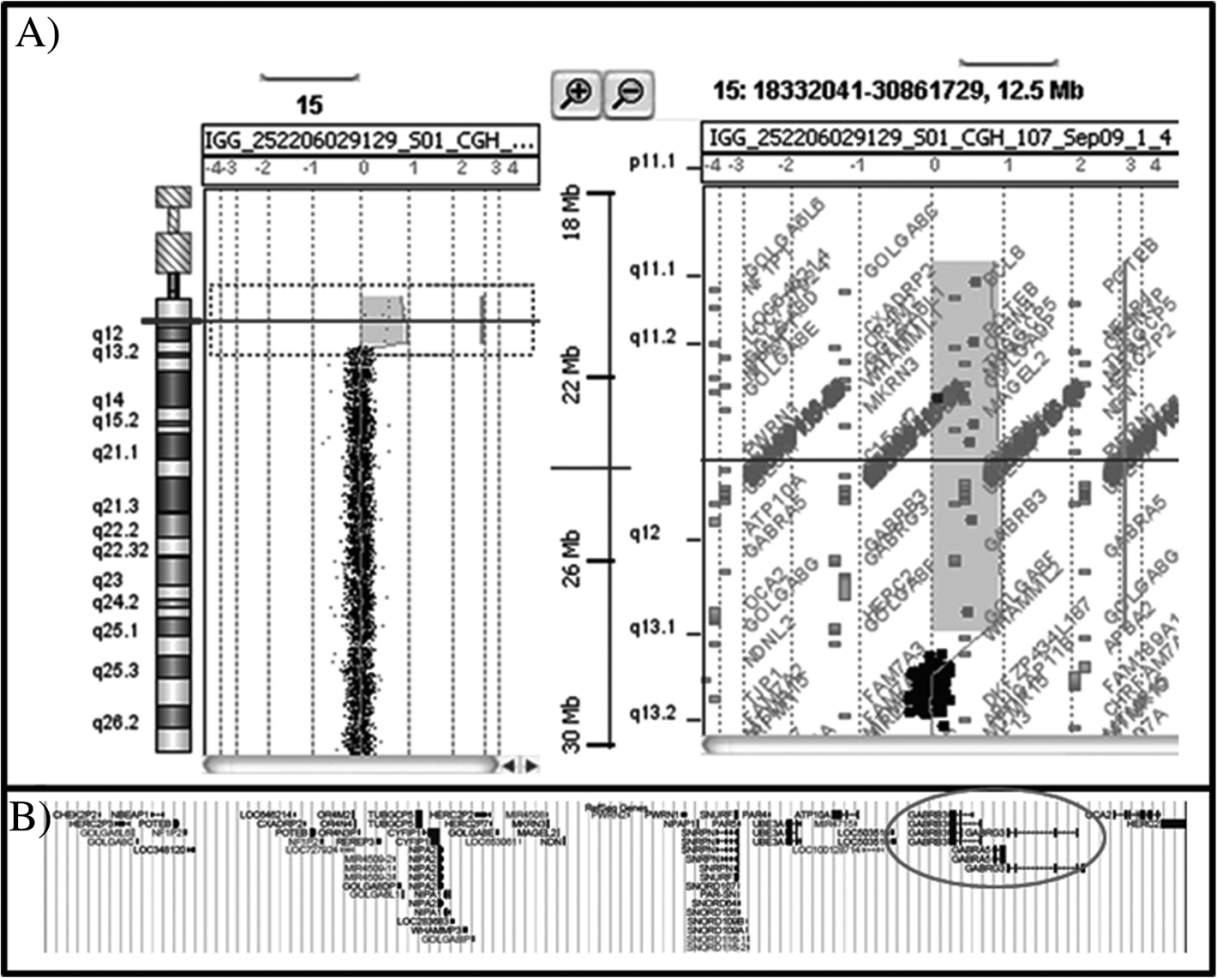
**(A) Array**-**CGH graphical overview of chromosome 15 duplication. **The 15q11.1q13.1 duplicated region extends between probes A_16_P02992133 (20,102,541 bp, first deleted) and A_16_P02998642 (28,535,051 bp, last deleted). (**B**) Gene content of the duplicated region. Genes *GABRB3*, *GABRG3*, and *GABRA5 *are circled.

The subject of this report is a 53-year-old woman with IDIC-15 with psychomotor slowing and moderately severe intellectual disability. Cytogenetic analysis evidenced a large supernumerary IDIC (15) in all the cells analyzed. To better characterize the abnormal chromosome, an array-CGH analysis was performed using the Agilent Human Genome CGH Microarray Kit G3 180 K platform (Agilent Technologies, Santa Clara, CA, USA), according to manufacturer’s protocol. Results showed a duplication encompassing 8.433 Mb between oligomers A_16_P02992133 (20,102,541 bp, first duplicated) and A_16_P02998642 (28,535,051 bp, last duplicated) (Figure 1A, B). The duplicated region harbors about 21 genes according to the UCSC Genome Browser (http://genome.ucsc.edu/; GRCh37/hg19, February 2009). Among these, in our opinion, *GABRB3* (gamma-aminobutyric acid A receptor, beta 3, MIM 137192), *GABRA5* (Homo sapiens gamma-aminobutyric acid A receptor, alpha MIM 137142) and *GABRG3* (gamma-aminobutyric acid A receptor, gamma 3 MIM 600233) genes could have a role in causing at least some phenotypic features of our proposita.

Her seizure disorder first appeared at age 24 when she developed complex partial seizures with staring and blanking-out episodes accompanied by stereotypical head turning or raising of the arms, without loss of muscle tone or falls that typically lasted a few seconds. At age 24 she also had an isolated generalized tonic-clonic seizure and was started on carbamazepine. Over the years, the complex partial seizures episodes became more frequent and when she was 34 years of age, Lamotrigine was added. As the frequency of the complex partial seizures increased, both drugs were kept at a dosing level to sustain serum medication levels at the higher limits of the norm. When she was 46 years old, oxcarbazepine was initiated and carbamazepine stopped.

At age 50 she had a fall and sustained severe head trauma that caused a large left parietal subdural hematoma and a small frontal contusion. After emergent surgical evacuation of the hematoma her seizures became more frequent with complex-partial episodes that occurred almost daily and often up to three times in a day that were of longer duration and accompanied by more prominent stereotypical hand and arm movements. An EEG could not be obtained as she did not tolerate the procedure and could not cooperate with the execution of the test. The daily doses of oxcarbazepine and lamotrigine were increased respectively to 1200 mg daily in two divided doses and 700 mg in three divided doses, with blood levels for both drugs sustained at the upper limits of the normal range. The change in dosing was only mildly effective, with seizure occurring four or five times per week, and she developed clear signs of drug toxicity with ataxia and imbalance and occasional nausea and vomiting. A year after the trauma, Pregabalin was added with doses that were slowly increased to 150 mg/day in three divided doses. Soon after she experienced a dramatic worsening of her seizures. Her complex partial crisis became more frequent and severe, with numerous episodes of staring accompanied by more prominent automatisms with raising of the arms above the head, forward bending of the trunk, head turning usually to the right, without falls. The episodes lasted up to 15–20 seconds and were followed by several minutes of obtundation and aphasic garbled speech. A new seizure pattern also developed, with atonic seizures characterized by sudden arrest and falls with altered level of consciousness and atonia. The dose of Pregabalin was then increased to the maximum tolerated dose of 300 mg daily in three divided doses, and her seizures became even more severe. Within days from the increase in dose she had an episode of four episodes of generalized tonic-clonic seizures followed in the following weeks by three more tonic-clonic seizures that lasted up to five minutes and were followed by prolonged post-ictal confusion, while continuing to experience frequent complex-partial seizures and atonic seizures. Most of the seizures were witnessed by one of the authors (PDR). She had also become cognitively more impaired, losing verbal capacity and often appearing obtunded and had become incontinent of urine and feces while remaining ataxic. Suspecting that the worsening of seizure frequency and the appearance of new seizure patterns were related to the addition of Pregabalin, the drug was tapered and eventually discontinued while Lacosamide was introduced and increased to a dose of 100 mg twice daily. Within two weeks of the drug change she experienced a dramatic improvement that has lasted now for over twelve months, with only one or two episodes of complex partial seizures per month with few and limited stereotypies, and no additional atonic crisis or generalized seizures. Her ataxia has also improved as the doses of Oxcarbazepine and Lamotrigine and have been decreased to 900 mg daily and 600 mg daily and she has resumed her routine life at home and in the day program that she attends, regaining her prior level of motor and intellectual performance and continence.

## Discussion

The IDIC-15 subject that we report had a dramatic worsening of her seizures when Pregabalin was added as third drug to ongoing treatment with lamotrigine and oxacarbazepine and almost complete seizure control when Pregabalin was stopped and lacosamide added.

Pregabalin is a gamma-aminobutyric acid (GABA) analog, structurally derived from GABA by the addition of an aliphatic side chain at the 3-position (S-(+)-3-isobutyl-GABA). Its anti-epileptic effect is mediated by the high-affinity binding to the α_2_δ subunit of the voltage-dependent calcium channel [14]. Although it is structurally related to GABA, pregabalin at therapeutic doses does not bind to GABA-A or GABA-B receptors, nor affects GABA uptake or degradation [15,16]. There is however evidence that in human motor cortex pregabalin produces a physiological effect similar to that of GABAergic drugs [17] and the drug affects the GABA transporter GAT-1, increasing neuronal GABA uptake [18].

There is no obvious explanation for the seizure worsening induced by pregabalin. In IDIC-15 both the intellectual disability and the seizure disorder are likely related to the abnormal GABA receptor morphology and function. The IDIC 15 chromosome in the subject we report, includes 8 Mb spanning 15q11-13.1 and results in 4 copies of the 21 genes found in this genomic interval, including genes encoding *GABRB3*, *GABRA5*, and *GABRG3*, and it is likely that the abnormal receptor configuration and distribution may modify the affinity of pregabalin for GABA receptors and its pharmacological effect. Interestingly, microdeletions in the 15q11.2 in the proximal BP1 – BP3 and BP2 – BP3 regions, have also been associated with a varied phenotype that include developmental and language delay, intellectual deficit, behavioral problems, autism, and seizures [19]. The mechanism leading to the neurological dysfunction is unknown, but it may be related to the deletion of the *NIPA1* and *CYFIP1*genes that are important in neurological development [19]. Other microdeletions in the 15q13.3 have been associated with idiopathic generalized epilepsy and other neuropsychiatric disorders including autism, schizophrenia, and autism [20]. Among the most common microdeletions are those affecting CHRNA7 in the BP4-BP5 region, the gene encoding the alpha 7 subunit of the nicotinic receptor [21], which can modulate GABA receptors’ function [22]. While it is possible that seizure activity can be related to abnormal cholinergic modulatory activity, in our case the duplication does not encompass the more distal BP4 – BP5 regions of the CHRNA 7 gene.

Increased of seizure activity and modification of seizures patterns have been reported with other drugs, including the GABAergic drugs vigabatrin [23] and tiagabine [24] and as well as drugs that are not GABAergic such as levetiracetam [25]. Gabapentin, a drug pharmacologically related to pregabalin, has been reported to induce life-threatening myoclonic status in a patient with benign adult familial myoclonic epilepsy [26]. While there may be other reasons that could explain the severe exacerbation of the seizure disorder after the introduction of pregabalin, the temporal coincidence and the worsening of the seizure disorder with increased dose of the drug suggest a direct effect of the drug. It is unlikely that the dramatic increase in seizure frequency and the appearance of different seizure patterns can be explained by a late effect of the trauma. While the subject’s stereotypical seizures had increased in frequency, the dramatic worsening occurred a year after the trauma, when pregabalin was introduced. Further, the progressive worsening of her seizures with increasing dose of pregabalin, the appearance of unusual seizure patterns, and the rapid improvement after pregabalin was removed and lacosamide introduced point to a pharmacological cause. It is however impossible to determine whether the dramatic improvement that she then experienced was due to the removal of pregabalin, the addition of lacosamide, or a combined effect.

## Conclusion

This case suggests that pregabalin and possibly other GABA analogs or gabaergic drugs should be used cautiously in individuals with IDIC- as the abnormal GABA receptor configuration and physiology could alter the pharmacological response to the drugs. Lacosamide, and presumably other ionic pump inhibitors may instead be safer and more effective in subjects with IDIC-15. This case also indirectly illustrates that people with IDIC-15 can maintain a stable level of neurological and intellectual function well into mature age if seizures are controlled. We believe that as with other rare diseases, even a single case experience may be helpful to clinicians treating seizures in individual with IDIC-15.

## Consent

Written informed consent was obtained from the patient's legal gurdian for publication of this Case report and any accompanying images. A copy of the written consent is available for review by the series editor of this journal.

## Competing interests

The authors declare that they have no competing interests.

## Authors’ contributions

ADR: study concept, manuscript preparation. AL: manuscript preparation and edit. MDR: genetic component of the study report and analysis. PDR: case history, manuscript revision. RR: manuscript preparation, editing and revison. GG: genetic testing. CB: epilepsy component of the study report and discussion. All authors read and approved the final manuscript.

## Pre-publication history

The pre-publication history for this paper can be accessed here:

http://www.biomedcentral.com/1471-2377/13/43/prepub
